# Determinants of health care costs in the senior elderly: age, comorbidity, impairment, or proximity to death?

**DOI:** 10.1007/s10198-017-0926-2

**Published:** 2017-08-30

**Authors:** Nisha C. Hazra, Caroline Rudisill, Martin C. Gulliford

**Affiliations:** 1 Department of Primary Care and Public Health Sciences, King’s College London, Guy’s Campus, 3rd Floor Addison House, London, SE1 1UL UK; 2 Department of Social Policy, London School of Economics and Political Science, London, UK; 3 National Institutes for Health Research Biomedical Research Centre at Guy’s and St Thomas’ National Health Service Foundation Trust, London, UK

**Keywords:** Health care costs, Electronic health records, Elderly, Ageing, Ecological fallacy, H41, H51, I10, I18

## Abstract

Ageing is assumed to be accompanied by greater health care expenditures but the association is also viewed as a ‘red herring’. This study aimed to evaluate whether age is associated with health care costs in the senior elderly, using electronic health records for 98,220 participants aged 80 years and over registered with the UK Clinical Practice Research Datalink and linked Hospital Episode Statistics (2010–2014). Annual costs of health care utilization were estimated from a two-part model; multiple fractional polynomial models were employed to evaluate the non-linear association of age with predicted health care costs while also controlling for comorbidities, impairments, and death proximity. Annual health care costs increased from 80 years (£2972 in men, £2603 in women) to 97 (men; £4721) or 98 years (women; £3963), before declining. Costs were significantly elevated in the last year of life but this effect declined with age, from £10,027 in younger octogenarians to £7021 in centenarians. This decline was steeper in participants with comorbidities or impairments; £14,500 for 80–84-year-olds and £6752 for centenarians with 7+ impairments. At other times, comorbidity and impairments, not age, were main drivers of costs. We conclude that comorbidities, impairments, and proximity to death are key mediators of age-related increases in health care costs. While the costs of comorbidity among survivors are not generally associated with age, additional costs in the last year of life decline with age.

**Electronic supplementary material:**

The online version of this article (doi:10.1007/s10198-017-0926-2) contains supplementary material, which is available to authorized users.

## Introduction

The senior elderly, aged 80 years and older, represent the fastest-growing age group in the majority of the developed world [19, 23] with the number of centenarians in the United Kingdom (UK) increasing by 65% over the past decade [24]. While increases in life expectancy and consequent rapid increases in the older age population are considered positive developments, the consequential future health care burden represents a leading concern for health services. Most commentaries incorporate an assumption that greater longevity and associated illness burden will be associated with substantial increases in health care costs.

A high proportion of lifetime health care costs incur towards the end of life [1, 9, 39], being associated with the management of terminal illness [12, 27] and the type of care received at the end of life [30]. This balance of costs across the life-course contributes to a potentially exaggerated assumption that increasing age itself is necessarily a driver of increased costs. The Organisation for Economic Co-Operation and Development’s (OECD) report on public spending in health and long-term care found that demographic change did not account for recent growth in public health care expenditures [25]. Between 1995 and 2009, public health spending grew by 4.3% per year on average in OECD countries, of which only 0.5% might be attributable to demographic developments [25].

Zweifel et al. [39] suggested that the proposed association of age with health care costs is a ‘red herring’. In their analyses, health care expenditures depended on remaining lifetime, and proximity to death, rather than calendar age [39]. Subsequent reports from the same authors and others have addressed methodological critiques [29] and confirmed the initial interpretation [13, 31, 35, 40]. However, while Zweifel et al. proposed that age is a ‘red herring’, Howdon and Rice [13] suggest that time to death is itself a ‘red herring’ that acts as a proxy for morbidity [13].

There is still presently insufficient evidence to support the ‘red herring’ claim in the senior elderly aged 80 years and older. Previous studies evaluating health care costs in the elderly evaluate younger elderly populations [5, 8, 14, 15, 18, 33] and few present data disaggregated by age category. In the senior elderly, age-related impairments (e.g., cognitive impairment, falls, fractures) become increasingly important, alongside multiple comorbidities [e.g., cancer, stroke, diabetes mellitus (DM)], but few studies have analyzed coded data for both impairments and morbidities [10]. Data for population sub-groups above 80 years are not widely reported and have not been analyzed separately in larger population-based samples. In smaller cohort studies, health care utilization and costs overall have been shown to increase with age, but some studies suggest that costs of medications, specialist visits, and surgical admissions may not increase beyond the age of 85 years [5, 34]. In the United States (US), Medicare spending between 2000 and 2001 increased with age until the mid-90s with spending decreasing in senior elderly years (95+) [21]. Another Medicare study reported average per-capita total health care expenditures in 2014 reaching a maximum at 97 years of age and per-capita spending being highest for those in their early 70s compared to older groups, mainly due to inpatient hospital-related spending [2]. Age has also previously been shown to have little predictive power on health care costs when controlling for life expectancy in the US, and the predictive power of life expectancy diminishes as health status variables are introduced [32]. Equivalent data have not been reported in large population-based cohorts of senior elderly in the UK.

The present study aimed to test the ‘red herring’ hypothesis in the senior elderly population. The study addresses an empirical gap in the literature concerning the trajectory of health care costs in the over-80s. We use primary care electronic health records (EHRs), with linked data for hospital utilization and drug prescribing, to analyze multiple age-related impairments, in addition to comorbidities, and to associate these with health care utilization and costs. We employed multivariable fractional polynomial models to estimate potential non-linearity in the association of age with health care costs, hypothesizing that in the senior elderly, aged 80 years and older, the independent effect of increasing age is not the main driver of health care costs, but rather the number of comorbidities, impairments, and proximity to death are.

## Methods

### Data source

Data were obtained from the Clinical Practice Research Datalink (CPRD), a nationally representative primary care database of EHRs in the UK containing anonymized patient records for approximately 7% of the UK population [11, 17]. Patients included in the database are broadly representative of the UK population [17] and EHR data including clinical diagnoses, consultations by type, and drug prescriptions have been shown to be valid in many studies [36]. The CPRD referral files also contain coded information from primary care EHRs concerning referrals to hospital and hospital discharge letters. The present study was restricted to general practices in England that participated in data linkage (372 practices in our sample) with secondary care data. Linked hospital utilization data were obtained from the Hospital Episode Statistics (HES) dataset [22] up to February 2016, providing information on hospital admissions. This study was approved through a protocol submitted to the Medicines and Health care Products Regulatory Agency (MHRA) Independent Scientific Advisory Committee (ISAC) for CPRD studies (Protocol No. 15_047).

### Sample

This research was part of a wider study on ageing. An age-stratified random sample was selected from the list of all patients registered at CPRD general practices. The sample was stratified by those who had their 80th, 85th, 90th, 95th, and 100th birthdays while registered with CPRD to provide over-representation of older age groups. The present analysis was restricted to those CPRD general practices in England that participated in HES data linkage in the most recent 5-year period, 2010–2014. The final sample comprised 98,220 participants aged 80 years and older, with linked HES records, and eligible person-time between 2010 and 2014.

### Costing analysis

Person-time at risk was calculated for each participant by year from 2010 to 2014. Person-time was further subdivided into time in the last 12 months of life (decedents) and all other time (survivors). Eligible person-time was also stratified by 5-year age group, gender, comorbidity category, and impairment category using the first record for each condition, as reported previously [10]. Comorbidities included: coronary heart disease (CHD), stroke, cancer, DM, hypertensive diseases, chronic respiratory diseases, musculoskeletal diseases, digestive diseases, and nervous system disorders. Age-related impairments included: cognitive decline and memory problems, dementia, depression, confusion and delirium, falls, fractures, hearing impairment, incontinence, mobility and gait problems, visual impairment, and musculoskeletal pain. For analysis, comorbidities and age-related impairments were grouped into the categories: none; one to three; four to six; and seven or more.

The bottom-up costing approach was implemented by identifying and enumerating all types of resources used and assigning a unit cost to each resource. Primary care contacts were enumerated from EHRs, including general practice (GP) consultations, emergency consultations, telephone consultations, home visits, and out-of-hours consultations. Data for all drug prescriptions issued in primary care were also analyzed. Hospital utilization, including inpatient hospital admissions, outpatient visits, day case visits, and accident and emergency (A&E) visits, were enumerated from referral records with linked HES data for inpatient admissions. Unit costs of health care utilization were obtained from standard reference sources including the Personal Social Services Research Unit (PSSRU) Costs of Health and Social Care 2015 report [26] for primary care utilization and the NHS reference costs [3] (Supplementary Table 1) for secondary care utilization. The total number of drugs prescribed from 2010 to 2014 were enumerated in CPRD and unit prescription costs were obtained by linking the Gemscript drug code for each prescription to item-specific costs from the RESIP Gemscript Code Dictionary (RESIP UK, Chertsey, Surrey, UK). All utilization analyses and costing of prescriptions were conducted using Stata Version 14.0.

### Two-part model

We modeled the association of annual health care costs with age group, gender, comorbidity category, impairment category, and proximity to death as well as interactions between these variables and costs. Proximity to death was represented using a dummy variable for person-time in the 12 months before death. A two-stage regression model was employed [4, 16]:

$$E\left( {Y |X} \right) = P\left( {Y > 0 |X} \right) \times E(Y|X,Y > 0),$$

where *P* is the probability of non-zero costs; *E* the expected value of; *Y* the cost of health care utilization per participant year; and *X* represents covariates of interest.

A probit model was employed to predict the probability of costs being incurred [6] allowing for the proportion of participants not utilizing services, which with an elderly population is low but still existent. A general linear model (GLM) [20] with log-link and gamma errors was employed to model health care expenditures, conditional on health care being utilized. The model incorporated the main effects of gender, dying (costs in last 12 months of life), comorbidity category and impairment category, as well as age and 5-year age group. Age was included as a categorical variable to allow for non-linearity of association [1]. We also included age as a continuous variable to account for differences in the distribution of age within age groups. All potential interaction terms were evaluated stepwise by comparing goodness-of-fit with or without the term. Due to the panel structure of the data, robust variance estimates were employed to account for correlation clustering of repeated observations on individual participants. The predicted costs of health care utilization were then estimated as the product of the predicted probabilities of health care being utilized and the predicted costs of utilization.

In order to further evaluate the association of age with model-predicted costs of health care utilization, we employed second-order fractional polynomial (FP) models [28]. The fractional polynomial approach systematically evaluates non-linearity by finding the best-fitting power transformation *x* ^*p*^, with *p* chosen from −2, −1, −0.5, 0, 0.5, 1, 2, 3, where *x* ^0^ represents log *x*. Second-order models take the form:

$$y = b_{0 + } b_{1} x^{p} + b_{2} x^{q} ,$$

where *q* is selected in the same manner as *p*. Models were fitted with age as a predictor of costs for sub-groups of gender, death (last 12 months of life) and comorbidity and impairment category. Models were fitted using the ‘mfp’ command in Stata version 14.0, with predicted values estimated using the ‘fracpred’ command. We did not incorporate FPs directly into the two-part model because the FP approach does not readily accommodate interaction terms.

## Results

There were 98,220 participants (54,014, 55%, women) contributing a total of 300,672 years of person-time to the analysis (Table 1). The proportion of person-time contributed by women increased from 49% at 80–84 years to 81% in centenarians. The age distribution of person-time was 31% at 80–84 years, 36% at 85–89 years, 24% at 90–94 years, 8% at 95–99 years and 1% for centenarians (Table 1). The proportion of person-time for decedents in the last 12 months of life was 4% at 80–84 years increasing to 24% in centenarians. The proportion of person-time associated with four or more comorbidities was 58% at 80–84 years but decreased from 95 years and above, while the proportion associated with four or more age-related impairments increased from 15% at 80–84 years to 31% in centenarians.

**Table 1:** Characteristics of sample

	80–84	85–89	90–94	95–99	100+	*p* value^a^
Person years	93,317	107,394	72,476	22,943	4542	
Female	45,439 (49)	55,922 (52)	43,528 (60)	17,567 (77)	3688 (81)	<0.001
Last year of life	3668 (4)	7883 (7)	9215 (13)	4686 (20)	1087 (24)	<0.001
Number of comorbidities						
0	1945 (2)	2168 (2)	1561 (2)	784 (3)	484 (11)	<0.001
1–3	36,842 (39)	39,344 (37)	26,679 (37)	9677 (42)	2132 (47)	0.003
4–6	51,591 (55)	62,138 (58)	42,018 (58)	11,981 (53)	1857 (41)	<0.001
7+	2939 (3)	3744 (3)	2218 (3)	500 (2)	69 (1)	<0.001
Number of impairments						
0	15,266 (16)	13,879 (13)	7513 (10)	2154 (9)	736 (16)	<0.001
1–3	64,405 (69)	70,766 (66)	44,722 (62)	13,070 (57)	2394 (53)	<0.001
4–6	12,944 (14)	21,151 (20)	18,452 (25)	6943 (30)	1275 (28)	<0.001
7+	702 (1)	1598 (1)	1788 (3)	775 (3)	137 (3)	<0.001

Figures are frequencies (column percents)

^a^Test for trend across age groups

The annual rate of home visits and out-of-hours consultations increased with age, but GP consultations and outpatient utilization declined significantly with age after 90 years and hospital inpatient utilization declined beyond 95 years of age (Table 2). Telephone consultations increased with age, declining after 99 years with lower annual utilization and cost rates among centenarians. The cost of all primary care services increased significantly with age from 80 up to approximately 95 or 99 years, before declining in the oldest age group, while secondary care service costs increased significantly from 80 to 90 years, reaching a plateau in nonagenarians and declining beyond 99 years; primary care costs peaked at 95–99 years (£676 per person year) and secondary care costs at 90–94 years (£2737 per person year). Annual prescription costs similarly increased with age from £578 per person year at 80–84 years to £712 at 90–94 years, with the lowest rate of prescription costs among centenarians (£526 per person year). The percent distribution across type of health care spending remained relatively constant across age groups; with primary care accounting for approximately 16% of cost, secondary care for 68% and prescriptions for 16% (Table 2).

**Table 2:** Age-stratified utilization, prescriptions, and costs by age-group and person-time, 2010–2014

	80–84 years	85–89 years	90–94 years	95–99 years	100+ years
	93,317	107,394	72,476	22,943	4542
General practice consultations					
Rate per person year	11.13	11.66	11.28	10.10	7.34
Cost per person year	£500.96	£524.83	£507.65	£454.52	£330.49
Telephone consultations					
Rate per person year	0.97	1.20	1.40	1.42	1.04
Cost per person year	£26.11	£32.29	£37.59	£38.29	£28.30
Home visits					
Rate per person year	0.40	0.77	1.34	2.03	2.02
Cost per person year	£35.73	£68.00	£118.92	£180.49	£179.33
Out-of-hours					
Rate per person year	0.02	0.03	0.04	0.05	0.05
Cost per person year	£0.84	£1.24	£1.56	£2.27	£2.18
Primary care cost (% total)	**£563.64 (16%)**	**£626.37 (16%)**	**£665.71 (16%)**	**£675.57 (17%)**	**£540.02 (19%)**
Prescriptions					
Rate per person year	68.2	80.1	88.8	86.7	63.9
Pres. cost (% total)	**£577.77 (16%)**	**£657.45 (16%)**	**£712.74 (17%)**	**£690.46 (18%)**	**£525.60 (18%)**
Inpatient episodes					
Rate per person year	0.81	0.91	0.91	0.86	0.61
Cost per person year	£2206.14	£2479.75	£2495.86	£2352.18	£1672.39
Outpatient visits					
Rate per person year	0.85	0.90	0.86	0.76	0.46
Cost per person year	£234.13	£248.57	£235.52	£207.59	£126.61
Day case episodes					
Rate per person year	0.01	0.01	0.004	0.002	0.001
Cost per person year	£3.99	£3.58	£2.80	£1.6	£0.48
Emergency visits					
Rate per person year	0.02	0.02	0.02	0.02	0.017
Cost per person year	£1.99	£2.42	£2.67	£3.08	£2.24
Secondary care cost (% total)	**£2446.26 (68%)**	**£2734.32 (68%)**	**£2736.84 (67%)**	**£2564.48 (65%)**	**£1801.70 (63%)**
Total cost	**£3587.66**	**£4018.14**	**£4115.29**	**£3930.51**	**£2867.33**

**Fig. 1:**
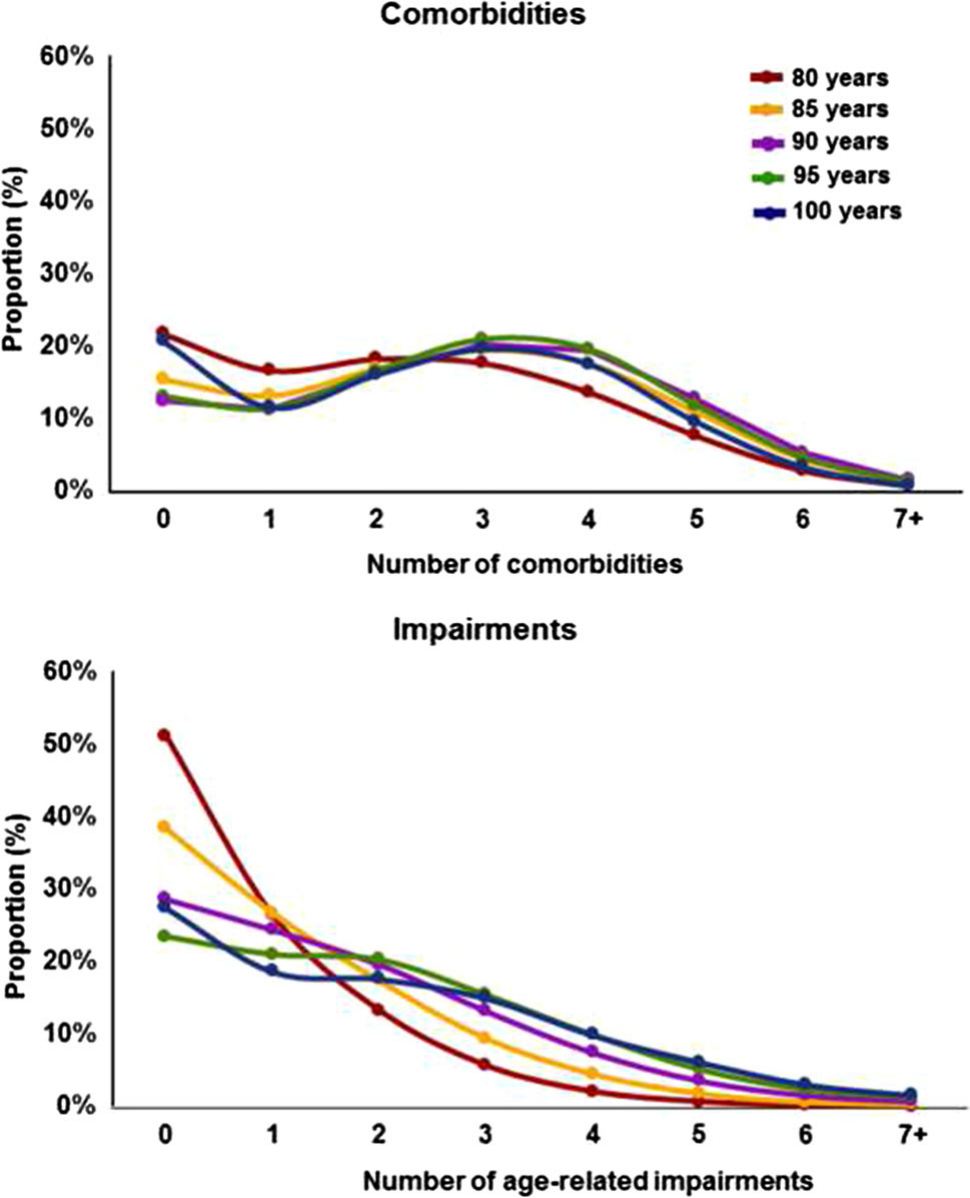
Proportion with different number of comorbidities and age-related impairments by age

The two-part regression model estimating predicted costs based on the cohort’s health service utilization is presented in Table 3. Higher coefficients associated with a covariate indicate a greater probability of utilizing health care in the probit model, or greater health care costs in the GLM. Women were more likely than men to use health care or to incur positive costs, as suggested in the probit model (coeff. 0.29, 95% CI 0.19–0.39, *p* < 0.001) (Table 3), but among those using health care, there was no significant difference in spending between men and women including all interaction terms (−0.12, −0.35 to 0.11, *p* = 0.289). The probability of using health care did not change with age, except for over-100s displaying a significantly lower probability of incurring costs compared to 80–84-year-olds (−0.94, −1.23 to −0.64, *p* < 0.001). Among those using health care, costs remained similar in all age-groups with non-significant coefficients in the GLM. With increasing comorbidity and impairment category, the probability of incurring positive costs increased in the probit model (2.27, 2.08–2.46, *p* < 0.001 for 7–9 comorbidities compared to none), as did the estimated costs among users of health care in the GLM (1.34, 1.10–1.58, *p* < 0.001 for 7–9 comorbidities compared to none). The proportion with comorbidities and age-related impairments are presented by age in Fig. 1. Proximity to death proved to be the strongest driver of high cost with the probability of using health care (0.79, 0.65–0.93, *p* < 0.001) and the cost of health care (1.46, 1.24–1.68, *p* < 0.001) being significantly higher in the last 12 months of life. The additional costs associated with dying declined substantially with age. There was a quantitatively important age-group interaction with proximity to death. This interaction term indicates that the probability of incurring health care costs increased with age among decedents, and provides justification for sub-group level presentation of costs. While there were significant increases in the probability of decedents using health care for each successive age-group (0.19, 0.11–0.26, *p* < 0.001 at 85–89 years; 1.74, 1.58–1.90, *p* < 0.001 at 100+ years), estimated costs among decedents using care declined with age-group, as shown in the GLM (−0.38, −0.55 to −0.20, *p* < 0.001 at 100+ years compared to 80–84 years).

**Table 3:** Two-part regression model for health care costs

Predictor	Probit model		GLM model	
	Coefficient (95% confidence interval)	*p* value	Coefficient (95% confidence interval)	*p* value
Age				
Single year	−0.04 (−0.05 to −0.04)	<0.001	0.001 (−0.003 to 0.006)	0.543
Gender				
Female	0.29 (0.19–0.39)	<0.001	−0.12 (−0.35 to 0.11)	0.289
Age group (years)				
80–84	Ref.		Ref.	
85–89	0.09 (−0.01 to 0.19)	0.089	0.35 (0.05–0.65)	0.024
90–94	0.04 (−0.09 to 0.17)	0.515	0.42 (0.18–0.67)	0.001
95–99	−0.09 (−0.27 to 0.09)	0.320	0.31 (0.02–0.60)	0.039
100+	−0.94 (−1.23 to −0.64)	<0.001	0.40 (−0.20 to 1.01)	0.192
Proximity to death				
Year before death (YD)	0.79 (0.65–0.93)	<0.001	1.46 (1.24–1.68)	<0.001
Age group × year before death (YD) interaction				
80–84.YD	Ref.		Ref.	
85–89.YD	0.19 (0.11–0.26)	<0.001	−0.10 (−0.18 to −0.02)	0.012
90–94.YD	0.40 (0.32–0.47)	<0.001	−0.23 (−0.31 to −0.16)	<0.001
95–99.YD	0.75 (0.66–0.85)	<0.001	−0.29 (−0.38 to −0.20)	<0.001
100+ .YD	1.74 (1.58–1.90)	<0.001	−0.38 (−0.55 to −0.20)	<0.001
Comorbidity category				
0	Ref.		Ref.	
1–3	1.55 (1.46–1.65)	<0.001	0.48 (0.25–0.71)	<0.001
4–6	2.25 (2.15–2.35)	<0.001	0.93 (0.70–1.16)	<0.001
7–9	2.27 (2.08–2.46)	<0.001	1.34 (1.10–1.58)	<0.001
Impairment category				
0	Ref.		Ref.	
1–3	0.40 (0.34–0.46)	<0.001	0.06 (−0.06 to 0.18)	0.305
4–6	0.54 (0.44–0.65)	<0.001	0.44 (0.31–0.56)	<0.001
7–10	0.75 (0.39–1.11)	<0.001	0.73 (0.53–0.93)	<0.001
Constant	3.53 (2.99–4.08)	<0.001	6.90 (6.46–7.35)	<0.001
Interactions				
Gender × age	*χ* ^2^ = 18.47, *df* = 4	<0.001	*χ* ^2^ = 6.23, *df* = 4	0.1826
Gender × MM	*χ* ^2^ = 35.51, *df* = 3	<0.001	*χ* ^2^ = 1.12, *df* = 3	0.7727
Gender × MI	*χ* ^2^ = 5.94, *df* = 3	0.1147	*χ* ^2^ = 7.96, *df* = 3	0.0469
Age × MM	*χ* ^2^ = 76.19, *df* = 12	<0.001	*χ* ^2^ = 60.44, *df* = 12	<0.001
Age × MI	*χ* ^2^ = 66.10, *df* = 12	<0.001	*χ* ^2^ = 30.46, *df* = 12	0.0024
YD × gender	*χ* ^2^ = 9.35, *df* = 1	0.0022	*χ* ^2^ = 0.00, *df* = 1	0.9871
YD × age	*χ* ^2^ = 630.85, *df* = 4	<0.001	*χ* ^2^ = 57.73, *df* = 4	<0.001
YD × MM	*χ* ^2^ = 259.55, *df* = 3	<0.001	*χ* ^2^ = 28.71, *df* = 3	<0.001
YD × MI	*χ* ^2^ = 66.28, *df* = 3	<0.001	*χ* ^2^ = 71.43, *df* = 3	<0.001

Table 4 outlines mean annual predicted costs of health care utilization by age subgroups of gender, proximity to death, comorbidity category and impairment category. Overall, annual costs of health care utilization increased from £3095 at 80–84 years to £4322 at 95–99 years, declining to £3698 among centenarians. A marginal increase in cost is observed with increasing age from 80  years in all sub-groups, followed by lower and declining costs in the later nonagenarian years up to 100 years and older. Costs among decedents in the 12 months before death were considerably higher compared to other years. For decedents in the last 12 months of life, mean annual predicted costs declined from £10,027 at 80–84 years to £7021 in centenarians, while remaining relatively constant with age among survivors, ranging from £2000 to £3000. In all age groups, costs increased with a greater number of comorbidities and impairments, with the largest increases among younger age-group decedents in the 12 months before death.

**Table 4:** Distribution of predicted costs of health care utilization by participant characteristics and age group

	80–84	85–89	90–94	95–99	100+
All participants	3095	3686	4081	4322	3698
Male	3295	4020	4600	4797	4030
Female	2882	3372	3721	4174	3625
Not last year	2579	2848	2948	2834	2004
Last year of life	10,027	9707	8677	7938	7021
Comorbidities [survivors]					
0	598	807	758	535	103
1–3	1796	2107	2209	2277	1728
4–6	3084	3273	3385	3371	2799
7+	5026	4995	5413	4728	4706
Comorbidities [decedents]					
0	3608	4856	4799	4123	2854
1–3	7123	7527	6966	6852	6380
4–6	11,200	10,576	9456	8798	7783
7+	14,308	12,752	11,848	9561	8950
Impairments [survivors]					
0	1802	1872	1670	1291	638
1–3	2481	2704	2754	2615	1971
4–6	3856	3833	3764	3586	2868
7+	5225	4916	4994	4336	3206
Impairments [decedents]					
0	8382	8012	6677	5930	8182
1–3	9650	9409	8487	7785	6994
4–6	11,808	10,734	9249	8381	6904
7+	14,500	12,273	11,002	9118	6752

Figures are UK £2014

The association of age with health care costs was further investigated in fractional polynomial (FP) models. In order to present the previously demonstrated significant interaction effects, FP models were fitted separately for sub-groups of gender, death and category of comorbidity, and age-related impairment. FP terms selected for age in each sub-group are presented in Table 5. First- and second-order terms selected for analysis of person-time among decedents in the last 12 months of life were generally inverse-squared terms, while cubic terms were selected for analysis of costs among survivors.

**Table 5:** First- and second-order term for age from fractional polynomial models with predicted costs of health care utilization as dependent variable, by participant characteristics

Group	First term	Second term
Men	X^3^ − 657.2 (*p* < 0.001)	X^3^ × ln(X) − 1421.4 (*p* < 0.001)
Women	X^3^ − 696.5 (*p* < 0.001)	X^3^ × ln(X) − 1519.7 (*p* < 0.001)
Surviving	X^3^ − 669.4 (*p* < 0.001)	X^3^ × ln(X) − 1451.8 (*p* < 0.001)
Died in year	X^−2^ − 0.0123 (*p* < 0.001)	X^−2^ × ln(X) − 0.0270 (*p* < 0.001)

	Surviving	Died	Surviving	Died
Number of morbidities				
0	X^−2^ − 0.0126 (*p* < 0.001)	X^−2^ − 0.0120 (*p* < 0.001)	X^−2^ × ln(X) − 0.0276 (*p* < 0.001)	X^−2^ × ln(X) − 0.0266 (*p* < 0.001)
1–3	X^3^ − 670.0 (*p* < 0.001)	X^−2^ − 0.0121 (*p* < 0.001)	X^3^ × ln(X) − 1453.2 (*p* < 0.001)	X^−2^ × ln(X) − 0.0267 (*p* < 0.001)
4–6	X^3^ − 668.0 (*p* < 0.001)	X^−2^ − 0.0123 (*p* < 0.001)	X^3^ × ln(X) − 1448.4 (*p* < 0.001)	X^−2^ × ln(X) − 0.0271 (*p* < 0.001)
≥7	X^3^ − 660.3 (*p* < 0.001)	Age − 88.90 (*p* < 0.001)	X^3^ × ln(X) − 1429.0 (*p* < 0.001)	–
Number of impairments				
0	X^−2^ − 0.0133 (*p* < 0.001)	X^3^ − 706.8 (*p* < 0.001)	X^−2^ × ln(X) − 0.0287 (*p* < 0.001)	X^3^ × ln(X) − 1545.7 (*p* < 0.001)
1–3	X^3^ − 662.6 (*p* < 0.001)	X^−2^ − 0.0124 (*p* < 0.001)	X^3^ × ln(X) − 1434.8 (*p* < 0.001)	X^−2^ × ln(X) − 0.0272 (*p* < 0.001)
4–6	X^3^ − 698.9 (*p* < 0.001)	X^0.5^ − 3.0 (*p* < 0.001)	X^3^ × ln(X) − 1525.7 (*p* < 0.001)	–
≥7	X^3^ − 725.2 (*p* < 0.001)	Age − 91.6 (*p* < 0.001)	X^3^ × ln(X) − 1592.2 (*p* < 0.001)	–

X refers to age/10

**Fig. 2:**
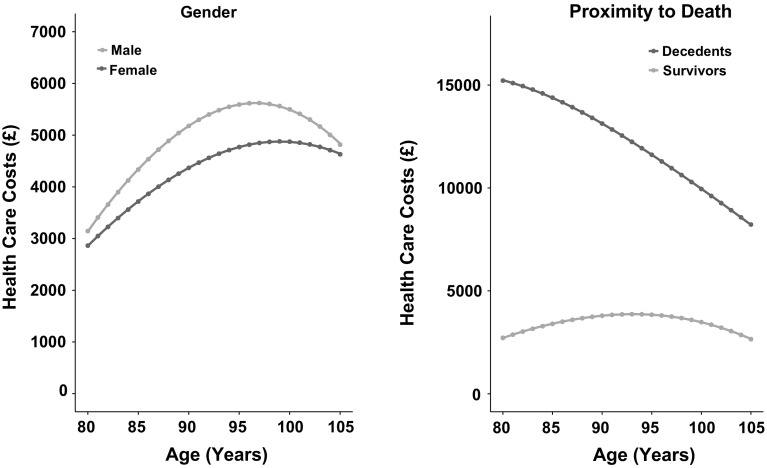
Predicted costs of health care utilization by year of age for sub-groups of gender and proximity to death. Estimates were predicted from multiple fractional polynomial model

**Fig. 3:**
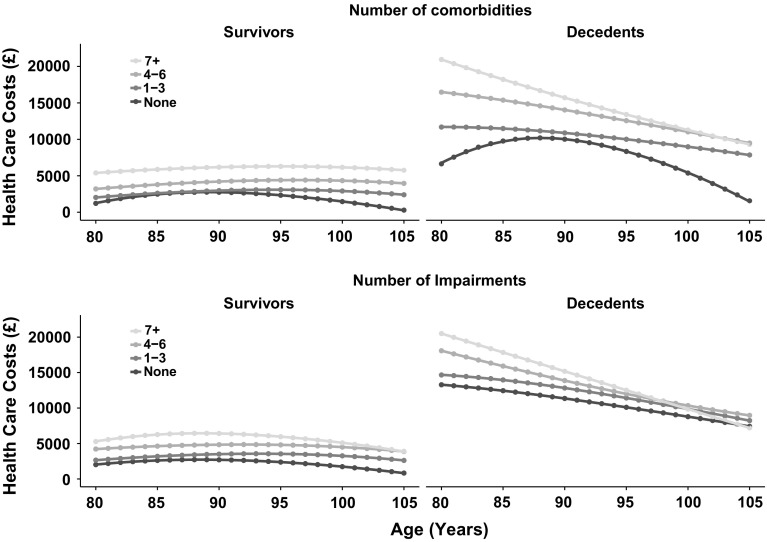
Predicted costs of health care utilization by year of age for sub-groups of proximity to death, comorbidity and impairment category. Estimates were predicted from multiple fractional polynomial model

Annual predicted costs of health care utilization increased from age 80 to age 97 (men) or 98 (women) (Fig. 2, left panel), before declining to age 105 years. Estimated costs by single year of age are presented in Supplementary Table 2. The effect of age differed for decedents and survivors (Fig. 2, right panel). Costs incurred among decedents in the last 12 months of life declined steeply with age, while among survivors costs tended to remain constant with age apart from a slight decline at the oldest ages. The data are presented disaggregated by sub-group of comorbidity and age-related impairment in Fig. 3. Among survivors, costs increased as the number of comorbidities and impairments increased but showed no consistent trend with age. Among decedents, costs decreased with age with the steepest declines observed in the highest categories of comorbidity or impairment. In the last 12 months of life, costs also were higher as the number of comorbidities increased in all age groups and were greater as the number of impairments increased in octogenarians and nonagenarians, but not in centenarians. For participants without comorbidity or impairment, age was more weakly associated with costs incurred by decedents in the last 12 months of life.

## Discussion

### Summary of findings

This study investigated associations between health care costs and age, proximity to death, comorbidity, and impairment among the senior elderly. The study presents novel data concerning the main drivers of health care costs in a large cohort of participants aged 80 years and older in the UK. Previous studies evaluating health care costs in the elderly evaluate younger elderly populations [5, 15, 33, 34] and age-stratified results beyond 80 years have rarely been presented but have unique features that deserve separate attention. At the population level, mean costs of health care utilization increased with age at least until the mid-nonagenarian years before declining in centenarians. The latter trend may result from a selection effect where the healthiest individuals survive to the oldest ages [10]. When controlling for proximity to death, comorbidity, and impairment, age was only weakly associated with costs, consistent with the ‘red herring’ claim. In the subgroup of participants who are in the last year of life, costs of health care utilization were negatively associated with age, rather than the almost universally expected positive association that Zweifel et al. have disputed. Declining costs with age among decedents was also reported in a primarily descriptive study of aggregate level data from the US [21], but represents a new finding in the UK. Among survivors, comorbidities and age-related impairments, rather than age, were associated with health care costs. The ‘red herring’ hypothesis, however, focuses on time to death rather than comorbidities, recognizing that the share of people close to death rises with age. This also applies to comorbidities and impairments; health care costs are a function of health status as the proportion with greater morbidity generally increases with age before declining beyond the mid-90s. Disentangling the independent effects of these characteristics is complex, some of which may be viewed as mediating variables. We argue that, when it comes to rising health care costs as populations age, our attention must start shifting towards subgroup-level characteristics of health status, as evaluated in our study, rather than age alone at a population level, particularly because time to death is difficult to quantitatively ascertain or predict.

### Comparison to existing literature

Population-based empirical studies concerning the complex relationship between health care costs, age, and health status are lacking in the senior elderly. Previous costing studies in the elderly have evaluated associations with health care costs and a variety of factors, but actual estimated cost figures in over-80s are scarce in the literature. Mean annual costs of health and social care have been reported at £7704 among over-70s discharged from an acute medical unit in England [8], a higher estimate than our figures, which is likely due to the inclusion of social care costs in the study and the severity of the sample’s health status. Existing research in the US has also reported average Medicare per-capita spending in 2011 more than doubling from $7566 at age 70 to $16,145 at age 96 before falling, with higher spending in mid-to-late 90s driven by spending on skilled nursing facilities, hospice care, and home health services [21]. When these services were excluded, per-capita spending peaked at age 89. Consistent with our data reporting males using more secondary care and females accounting for more prescription costs, a Canadian study described males aged 65 years and older using more specialist care, emergency room visits, and surgical admissions, with females using significantly more medications and attending more GP visits [34].

Compared to younger elderly individuals aged 65–79, octogenarians have also been reported to attend significantly more GP visits (4.4 visits per person year) and use more medications (8.2 per person year) [34], which is supported by our results showing increasing rates of GP visits with age from 80 to 90 years. Our study supplements this with new data illustrating a decreasing rate of GP visits beyond 90 years of age, with a concurrent increase in home visits, out-of-hours and telephone consultations among nonagenarians and centenarians. Modestly rising health care costs from 80 years and declining costs beyond 95 years between 2010 and 2014 observed in our study show consistent patterns with an older American study reporting increases in any physician contact from 70 years of age up to 85 years, followed by a lower probability of any physician contact beyond 85 years between 1993 and 1995 [5]. The study, however, focused primarily on ethnic and racial disparities in health care utilization, and our study is the first using UK data to evaluate proximity to death or the relationship between costs and other health-related covariates compared to age.

The fixed percent breakdown of primary care, secondary care, and prescription costs in our cohort across age groups and type of condition represents a new finding among over-80s and is relatively consistent with an English study identifying patient-level health and social care costs among over-70s reporting an 11% and 76% cost contribution from primary and secondary care, respectively [8]. Franklin et al. however, did not stratify by age group or include prescription costs. The study included social care costs in addition to health care costs, reporting a social care cost contribution of 10% for all health and social care costs.

A review reporting health care costs having a curvilinear positive, nearly exponential, relationship with multiple chronic conditions in younger elderly groups [14] is consistent with our findings in the senior elderly illustrating a positive relationship between level of comorbidity and health care costs. However, our study is the first to report data for over-80s on the relationship between costs and a multitude of age-related impairments, including cognitive impairment, falls, fractures, hearing impairments, and dementia, showing increasing impairment and comorbidity being an even greater driver of costs compared to age and gender. It is established in the literature that the cost of health and social care among individuals at the end of life is significant, and much of this cost is borne by informal care givers [27, 33]. Our analysis indicates that high costs of care at the end of life also holds true in a health care setting alone and we provide new evidence regarding the differing effect of age, comorbidity, and impairment on costs among decedents in the last 12 months of life compared to survivors in an English dataset.

### Strengths and limitations

This study’s findings are strengthened by its use of a large nationally representative sample of senior elderly, enabling us to analyze longitudinal utilization data for up to 7% of the UK population. Use of primary care EHRs facilitated analysis by age group for a wide range of chronic conditions and impairments in a large sample. Approximately 98% of the UK population will be registered with a family practice, ensuring our results are population based.

However, several limitations to our study must be acknowledged. We did not have access to data on participants’ place of residence and therefore could not characterize individuals based on whether they lived in a residential care home or in a private residence. Analysis from our previous study on centenarians revealed difficulties in ascertaining place of residence in CPRD [10]. Participants could be moving into residential care homes, which may affect recording of utilization patterns in CPRD by potentially underestimating health care costs for this population. Institutionalization has been shown in the literature to make up a large part of health care costs for the elderly [15], with most spending among high-cost users coming from institutional care [37], but we did not have access to social care data outside CPRD. The present analysis might underestimate total end of life costs for care from a societal perspective but still accurately captures the effects on the health care system itself. An understanding of social care utilization would be a particularly valuable addition to this analysis, in order to provide a complete picture of all health and social care costs among the senior elderly and how they are borne by each sector. Further research might also examine the relationship between type of disease, proximity to death, and age, as in Wong et al. [38].

Our study still provides important new figures from a comprehensive health care perspective on the complex relationship between health care costs and its potential determinants, in a severely understudied and rapidly growing group of senior elderly in the UK. We most importantly report new findings on the polarized association between age and health care costs by proximity to death.

Our calculation of hospital inpatient episodes in HES represents an inpatient consultation in the care of one consultant. This may be recorded as two episodes even if the patient is in the hospital for one problem. For example, hip replacement may be recorded as an orthopedic episode and a geriatric episode. This may slightly overestimate our calculations for inpatient hospital consultations. It should be noted that prescriptions recorded in CPRD likely reflect mostly primary care prescriptions as prescriptions given in the hospital are generally for a short duration. However, these hospital prescriptions are likely to be bundled into the consultation cost through the NHS tariffs and any new ongoing prescriptions started in the hospital would be continued by primary care prescribers. Therefore, our data picks up these prescription costs through an initial brief upfront cost in the hospital.

We performed additional robustness checks considering other potential model specifications. The possibility of using a finite mixture of GLMs was considered, used previously by Eckardt et al., but was not adopted due to little improvement in reported goodness-of-fit [7]. In addition, we were interested in the relative and independent effects of comorbidity and impairment category, proximity to death and age on health care costs rather than understanding identifiable components of various comorbidity groups. Using a negative binomial regression was also explored, but was deemed not appropriate for our data due to its suitability for over-dispersed count data.

### Conclusions

The findings of this study indicate that impairment and comorbidity are stronger drivers of health care costs than increased age alone, with proximity to death being the strongest predictor of high costs. The importance of proximity to death is highlighted in our findings through the contrasting relationship between age and cost contingent on proximity to death. While our population-level analysis supports the ‘red herring’ hypothesis, we also present declining costs with age among decedents in the last 12 months of life, demonstrating an unconventionally negative age gradient at this subgroup level. We also highlight the need for a shift from a population-based emphasis on age towards a more stratified subgroup-level approach that further recognizes health status when evaluating health care costs in over-80s. These new findings will be essential in helping inform policy-makers responsible for priority setting and planning for the health care needs of an ageing population. More research is required to further understand the components of health care costs in the months before death, with the incorporation of social care and institutional costs. Public health efforts will be crucial in reducing high levels of age-related impairments and chronic morbidities, and their associated costs, in addition to better managing these conditions in the senior elderly.

## Electronic supplementary material

Below is the link to the electronic supplementary material.

Supplementary material 1 (DOCX 31 kb)
