# Supplementary material for: Determinants of health care costs in the senior elderly: age, comorbidity, impairment, or proximity to death?
Source: Eur J Health Econ. 2017 Aug 30;19(6):831–42. doi: 10.1007/s10198-017-0926-2 (PMC6008359; doi:10.1007/s10198-017-0926-2)
Supplement: Supplementary file 1 — Supplementary material 1 (DOCX 31 kb) [file 10198_2017_926_MOESM1_ESM.docx]

**Supplementary Table 1: Unit costs for primary and secondary care consultations.**

| Consultation type | Unit cost | Source |
| --- | --- | --- |
| Primary Care |  |  |
| GP | £45 | PSSRU 2015 |
| Telephone consultation | £27 | PSSRU 2015 |
| Home visit | £88.92^1^ | PSSRU 2015 |
| Out-of-hours visit | £45 | PSSRU 2015 |
| Secondary Care |  |  |
| Day case | £721 | NHS Reference Costs 2014-15 |
| Inpatient^2^  Elective  Non-elective | £2,729.64  £3,573  £1,565 | NHS Reference Costs 2014-15 |
| Outpatient | £275 | NHS Reference Costs 2014-15 (service code 430 – Geriatric Medicine^3^) |
| Accident & Emergency (A&E) | £132 | NHS Reference Costs 2014-15 |

^1^Based on 11.4 minutes contact time plus 12 minutes travel time at rate of £3.80/minute, ^2^Weighted average based on 58% elective and 42% non-elective (GSTT figures, personal communication with Bryn Williams). Excluding excess bed days. ^3^From National Schedule of Reference Costs – Year 2014-15: Consultant Led Non-Admitted Face to Face Attendance, First.

| Age | Male | Female | Died | Living | No comorbidity | | 7+ comorbidities | | No impairments | | 7+ impairments | |
| --- | --- | --- | --- | --- | --- | --- | --- | --- | --- | --- | --- | --- |
|  |  |  |  |  | Died | Living | Died | Living | Died | Living | Died | Living |
| 80 | 2,972 | 2,603 | 10,216 | 2,436 | 3,400 | 539 | 14,715 | 4,874 | 9,011 | 1,735 | 14,827 | 5,031 |
| 81 | 3,153 | 2,736 | 10,159 | 2,517 | 3,721 | 602 | 14,446 | 4,925 | 8,788 | 1,792 | 14,489 | 5,064 |
| 82 | 3,327 | 2,863 | 10,086 | 2,592 | 3,992 | 654 | 14,177 | 4,971 | 8,572 | 1,833 | 14,151 | 5,089 |
| 83 | 3,492 | 2,985 | 9,999 | 2,661 | 4,218 | 696 | 13,908 | 5,014 | 8,363 | 1,861 | 13,814 | 5,106 |
| 84 | 3,649 | 3,101 | 9,899 | 2,722 | 4,401 | 728 | 13,638 | 5,052 | 8,161 | 1,875 | 13,476 | 5,114 |
| 85 | 3,797 | 3,211 | 9,787 | 2,777 | 4,545 | 752 | 13,369 | 5,085 | 7,967 | 1,878 | 13,138 | 5,114 |
| 86 | 3,936 | 3,314 | 9,665 | 2,823 | 4,652 | 767 | 13,100 | 5,114 | 7,782 | 1,871 | 12,800 | 5,105 |
| 87 | 4,064 | 3,412 | 9,534 | 2,862 | 4,727 | 774 | 12,831 | 5,137 | 7,605 | 1,853 | 12,463 | 5,087 |
| 88 | 4,183 | 3,502 | 9,393 | 2,893 | 4,770 | 775 | 12,562 | 5,156 | 7,437 | 1,826 | 12,125 | 5,059 |
| 89 | 4,291 | 3,585 | 9,245 | 2,915 | 4,784 | 769 | 12,292 | 5,169 | 7,279 | 1,791 | 11,787 | 5,022 |
| 90 | 4,388 | 3,661 | 9,090 | 2,929 | 4,772 | 757 | 12,023 | 5,176 | 7,131 | 1,748 | 11,449 | 4,974 |
| 91 | 4,473 | 3,729 | 8,928 | 2,933 | 4,726 | 740 | 11,754 | 5,178 | 6,993 | 1,698 | 11,112 | 4,916 |
| 92 | 4,547 | 3,789 | 8,761 | 2,928 | 4,677 | 717 | 11,485 | 5,173 | 6,865 | 1,642 | 10,774 | 4,847 |
| 93 | 4,608 | 3,840 | 8,589 | 2,913 | 4,598 | 690 | 11,216 | 5,163 | 6,749 | 1,579 | 10,436 | 4,767 |
| 94 | 4,657 | 3,883 | 8,412 | 2,889 | 4,499 | 659 | 10,946 | 5,146 | 6,645 | 1,511 | 10,098 | 4,675 |
| 95 | 4,692 | 3,917 | 8,230 | 2,854 | 4,382 | 624 | 10,677 | 5,123 | 6,553 | 1,438 | 9,760 | 4,572 |
| 96 | 4,713 | 3,942 | 8,046 | 2,808 | 4,284 | 585 | 10,408 | 5,093 | 6,473 | 1,360 | 9,423 | 4,456 |
| 97 | 4,721 | 3,957 | 7,858 | 2,752 | 4,100 | 542 | 10,139 | 5,056 | 6,406 | 1,279 | 9,085 | 4,328 |
| 98 | 4,714 | 3,963 | 7,667 | 2,684 | 3,938 | 497 | 9,870 | 5,012 | 6,352 | 1,193 | 8,747 | 4,187 |
| 99 | 4,692 | 3,958 | 7,474 | 2,604 | 3,763 | 449 | 9,600 | 4,960 | 6,312 | 1,104 | 8,409 | 4,033 |
| 100 | 4,655 | 3,943 | 7,279 | 2,513 | 3,576 | 398 | 9,331 | 4,901 | 6,286 | 1,012 | 8,072 | 3,866 |
| 101 | 4,601 | 3,917 | 7,082 | 2,410 | 3,378 | 345 | 9,062 | 4,834 | 6,275 | 916 | 7,734 | 3,685 |
| 102 | 4,532 | 3,879 | 6,883 | 2,293 | 3,170 | 290 | 8,793 | 4,760 | 6,279 | 819 | 7,396 | 3,489 |
| 103 | 4,445 | 3,831 | 6,683 | 2,164 | 2,954 | 233 | 8,524 | 4,676 | 6,299 | 719 | 7,058 | 3,279 |
| 104 | 4,341 | 3,771 | 6,483 | 2,022 | 2,729 | 174 | 8,255 | 4,585 | 6,334 | 617 | 6,720 | 3,054 |
| 105 | 4,220 | 3,698 | 6,281 | 1,866 | 2,496 | 113 | 7,985 | 4,485 | 6,386 | 513 | 6,383 | 2,814 |
|  |  |  |  |  |  |  |  |  |  |  |  |  |

**Supplementary Table 2: Predicted annual costs of health care utilisation by year of age, gender, comorbidity, impairments and whether in last 12 months of life. Figures are UK£ 2014.**
